# The Role of Systematic Reviews in Pharmacovigilance Planning and Clinical Trials Authorisation Application: Example from the SLEEPS Trial

**DOI:** 10.1371/journal.pone.0051787

**Published:** 2013-03-15

**Authors:** Carrol Gamble, Andrew Wolf, Ian Sinha, Catherine Spowart, Paula Williamson

**Affiliations:** 1 Clinical Trials Research Centre, University of Liverpool, Liverpool, Merseyside, United Kingdom; 2 Paediatric Intensive Care Unit, Bristol Royal Children's Hospital, Bristol, United Kingdom; 3 Respiratory Unit, Alder Hey Children's NHS Foundation Trust, Liverpool, Merseyside, United Kingdom; Universidad Peruana Cayetano Heredia, Peru

## Abstract

**Background:**

Adequate sedation is crucial to the management of children requiring assisted ventilation on Paediatric Intensive Care Units (PICU). The evidence-base of randomised controlled trials (RCTs) in this area is small and a trial was planned to compare midazolam and clonidine, two sedatives widely used within PICUs neither of which being licensed for that use. The application to obtain a Clinical Trials Authorisation from the Medicines and Healthcare products Regulatory Agency (MHRA) required a dossier summarising the safety profiles of each drug and the pharmacovigilance plan for the trial needed to be determined by this information. A systematic review was undertaken to identify reports relating to the safety of each drug.

**Methodology/Principal Findings:**

The Summary of Product Characteristics (SmPC) were obtained for each sedative. The MHRA were requested to provide reports relating to the use of each drug as a sedative in children under the age of 16. Medline was searched to identify RCTs, controlled clinical trials, observational studies, case reports and series. 288 abstracts were identified for midazolam and 16 for clonidine with full texts obtained for 80 and 6 articles respectively. Thirty-three studies provided data for midazolam and two for clonidine. The majority of data has come from observational studies and case reports. The MHRA provided details of 10 and 3 reports of suspected adverse drug reactions.

**Conclusions/Significance:**

No adverse reactions were identified in addition to those specified within the SmPC for the licensed use of the drugs. Based on this information and the wide spread use of both sedatives in routine practice the pharmacovigilance plan was restricted to adverse reactions. The Clinical Trials Authorisation was granted based on the data presented in the SmPC and the pharmacovigilance plan within the clinical trial protocol restricting collection and reporting to adverse reactions.

## Introduction

Adequate sedation is crucial to the management of children requiring assisted ventilation on the Paediatric Intensive Care Unit (PICU). The level of sedation of post-operative infants has been shown to impact on subsequent morbidity, and even mortality [1], [2], [3]. Most critically ill children will require potent analgesic and sedative drugs to facilitate artificial ventilation, alleviate pain, prevent discomfort from procedures, and to prevent distress from the presence of unfamiliar personnel and from the high level of background noise, which can disturb natural sleeping patterns [4]. However, while under-sedation can result in significant morbidity, over- sedation can also be associated with distressing or dangerous adverse effects, which may be difficult to assess in critically ill children [5]. The SLEEPS trial (www.controlled-trials.com/ISRCTN02639863), a prospective multi-centre randomised, double-blind, equivalence study was designed to compare clonidine and midazolam as intravenous sedative agents in critically ill children requiring mechanical ventilation.

Midazolam, a benzodiazepine derivative, is the most commonly used sedative in critically ill children, both in the UK and abroad [6]. For sedation of the critically ill child it is usually given in combination with an opioid by intravenous infusion at doses between 50–300 micrograms/kg/hr [7]. It can also be administered via subcutaneous infusion, or via intravenous bolus. Acute effects of midazolam exposure through continuous infusion include adverse effects of the drug on the cardiovascular system [8], [9], while continuous exposure to midazolam over several days results in rapid development of tolerance and subsequent withdrawal phenomena [1].

Clonidine is an α-2 adrenergic agonist which has antihypertensive, analgesic and sedative effects. In recent years, the drug has become more frequently used as a sedative in critically ill children and is usually administered with an opioid by intravenous infusion or orally.

Midazolam and clonidine are used widely within paediatric intensive care but are not licensed for that use. As the conditions of use in the clinical trial differed from those licensed, the Clinical Trials Authorisation application to the Medicines and Healthcare products Regulatory Agency (MHRA) required that the Summary of Product Characteristics (SmPC) for its licensed use be complemented with a summary of relevant data supporting its use in the proposed clinical trial. The trial aimed to open to recruitment in 2009 therefore we aimed to include safety data up to 2008.

Within the application for funding for the SLEEPS trial the conclusions from a systematic review [10] were used to justify the need for the new trial. The need to use systematic reviews to avoid unnecessary new research is clear but research conducted on using systematic reviews in the design and planning of a new trial have indicated that their potential is not being optimised [11], [12], [13], [14].

The aim of this paper is to describe the systematic review of safety data included within the Clinical Trials Authorisation application associated with the continuous infusion of midazolam or clonidine as sedation for neonates, infants and older children requiring mechanical ventilation and to illustrate how this informed the pharmacovigilance plan for the SLEEPS trial.

## Methods

All authors contributed to and agreed the protocol. Due to the variety of study designs, anticipated lack of direct comparisons, and required focus on safety profiles of each drug individually we did not plan to combine results within a meta-analysis or test for publication bias.

### Criteria for considering studies for the review

We included Randomised Controlled Trials (RCTs), Controlled Clinical Trials (CCTs), observational studies and case reports or case series.

We included only studies assessing the safety of continuous intravenous infusion of midazolam or clonidine when used as sedation for mechanically ventilated children under the age of eighteen years. For the literature search the age limit was set to allow variability in definition of paediatric. Because this review targeted safety, we included all studies which described the active or passive monitoring of adverse effects in the methods and/or the absence or presence of adverse effects in the results. We included studies administering midazolam or clonidine either on their own or with a concomitant opioid, reflecting routine clinical practice. We excluded studies assessing the use of midazolam or clonidine via any route other than a continuous intravenous infusion. We excluded studies assessing the use of midazolam as sedation for procedures, or as an anticonvulsant.

### Identification of studies

Prior to conducting the literature search to our knowledge there were no relevant published systematic reviews for clonidine and four relevant published systematic reviews [1], [10], [15], [16] for midazolam. Of these reviews, two related only to the use of midazolam in neonates [10], [15] and two were restricted to withdrawal symptoms [1], [16]. The Cochrane review assessing intravenous midazolam in neonates had been updated in 2002 [10].

MEDLINE was searched in September 2008. The search strategy is provided in Table 1. Two reviewers (IS and CS) independently screened each abstract identified and any queries were discussed with AW. Full texts of potentially eligible studies were obtained and their references screened to identify any additional eligible studies.

**Table 1:** Search Strategy.

Database: Ovid MEDLINE(R) <1950 to August Week 4 2008>
Search Strategy:
1 child$.mp. or child/(1500744)
2 (paediatric$ or pediatr$).mp. (163973)
3 infant/or infan$.mp. (864884)
4 young$.mp. (304417)
5 toddler.mp. (1117)
6 bab$.mp. (54771)
7 child,preschool/(625996)
8 (preschool or pre-school).mp. (627837)
9 adolesc$.mp. or adolescent/(1304128)
10 teenage$.mp. (10507)
11 youth$.mp. (21868)
12 or/1–11 (2734500)
13 (intensive care or critical care).mp. (90585)
14 intensive care units, pediatric.mp. (2630)
15 intensive care units, neonatal.mp. (6648)
16 picu.mp. (929)
17 p*ediatric intensive care.mp. (3149)
18 “critically ill”.mp. (16329)
19 ventilated.mp. (17171)
20 ventilat$.mp. (106904)
21 respiration, artificial.mp. (30372)
22 positive pressure respiration.mp. (13060)
23 intermittent positive pressure breathing.mp. (943)
24 intermittent positive pressure ventilation.mp. (2397)
25 (high frequency ventilation or high frequency oscillation).mp. (1912)
26 high frequency positive pressure ventilation.mp. (127)
27 (hfv or hfov).mp. (764)
28 high frequency jet ventilation.mp. (1084)
29 airway pressure release ventilation.mp. (78)
30 aprv {No Related Terms} (56)
31 continuous mandatory ventilation.mp. or continuous mandatory ventilation/(7)
32 cmv.mp. (14725)
33 intermittent mandatory ventilation.mp. (529)
34 (synchron$ intermittent mandatory ventilation or simv).mp. (206)
35 (pressure suppoprt ventilation or psv).mp. (1254)
36 (extracorporeal membrane oxygenation or ecmo or oxygenation, extracorporeal membrane).mp. (4039)
37 or/13–36 (223297)
38 (intravenous or iv).mp. (467655)
39 parenteral.mp. (63557)
40 inject$.mp. (556405)
41 infus$.mp. (224106)
42 (infus$ or infusions, intravenous).mp. (224106)
43 $venous.mp. (139547)
44 or/38–43 (1144844)
45 midazolam.mp. (7964)
46 midazolam hydrochloride.mp. (64)
47 midazolam maleate.mp. (26)
48 (dormicum or flormidal or versed or hypnovel or dormonid).mp. (496)
49 benzodiazepine.mp. or benzo$/(15817)
50 or/45–49 (22992)
51 (clonidine dihydrochloride or clonidine hydrochloride or clonidine monohydrobromide or clonidine monohydrochloride).mp. (200)
52 (clofelin or clopheline or dixarit or gemiton or hemiton or catapres or catapres*an or clophazolin or colfenil or isoglaucon or klofelin or klonefil or m-5041t or st-155).mp. (464)
53 (clonidine or clonidin$).mp. (15383)
54 or/51–53 (15506)
55 37 and 44 and 12 (8661)
56 50 and 55 (288)
57 53 and 55 (16)

Authors of studies that did not report but may have collected relevant data were not contacted due to time constraints to submit the CTA application. An assessment was made about the likelihood of outcome reporting bias within those studies [17]. For studies that included data for adults and children, authors were contacted to obtain data for children alone.

The Summary of Product Characteristics (SmPC) were obtained (midazolam from F.Hoffmann-La Roche Ltd as midazolam was originally licensed by this company; clonidine from Boehringer Ingelheim UK sole UK manufacturer).

Information was requested from the MHRA under the Freedom of Information Act regarding reports of midazolam and clonidine being used as a sedative in children. The age limit was set to be 16 years, the upper age limit planned for the SLEEPS trial.

### Data extraction

Data extraction was conducted by IS, CS, and CG. All extracted data were cross checked:

1. Study characteristics (design, age of participants, dose and duration of midazolam or clonidine therapy, concomitant sedative or opiate therapy);
2. Any data relating to the safety of these drugs, especially changes in blood pressure or heart rate on induction or cessation of midazolam or clonidine, withdrawal signs and symptoms, and negative effects on other organ function. The adverse effects were subsequently categorised into those relating to cardiovascular effects, withdrawal effects, and any other effects;

### Assessment of methodological quality

For each RCT and observational study we evaluated how rigorously the adverse event data were ascertained by assessing whether adverse effects were actively or passively monitored. We also determined how well the methods were described and whether or not the adverse effects of interest were defined a priori. We examined each study to see if the authors of the study had assessed the likelihood that adverse events were related to the use of medication, in other words whether they were considered to be Adverse Drug Reactions (ADRs). If an assessment of causality was made, we examined whether this was done using an existing, validated tool, such as that described by Naranjo [18]. We also assessed whether authors distinguished between midazolam or clonidine and a concomitant medication as being the cause of a suspected ADR.

In addition, for each RCT we assessed the adequacy of randomisation, allocation concealment and masking of interventions, and in order to assess the quality of reporting we evaluated whether all children who had received midazolam or clonidine were included in the safety analysis. We made no assessment of the quality of case reports or case series, other than whether or not causality had been assessed.

## Results

### Results of the search

**Figure 1:**
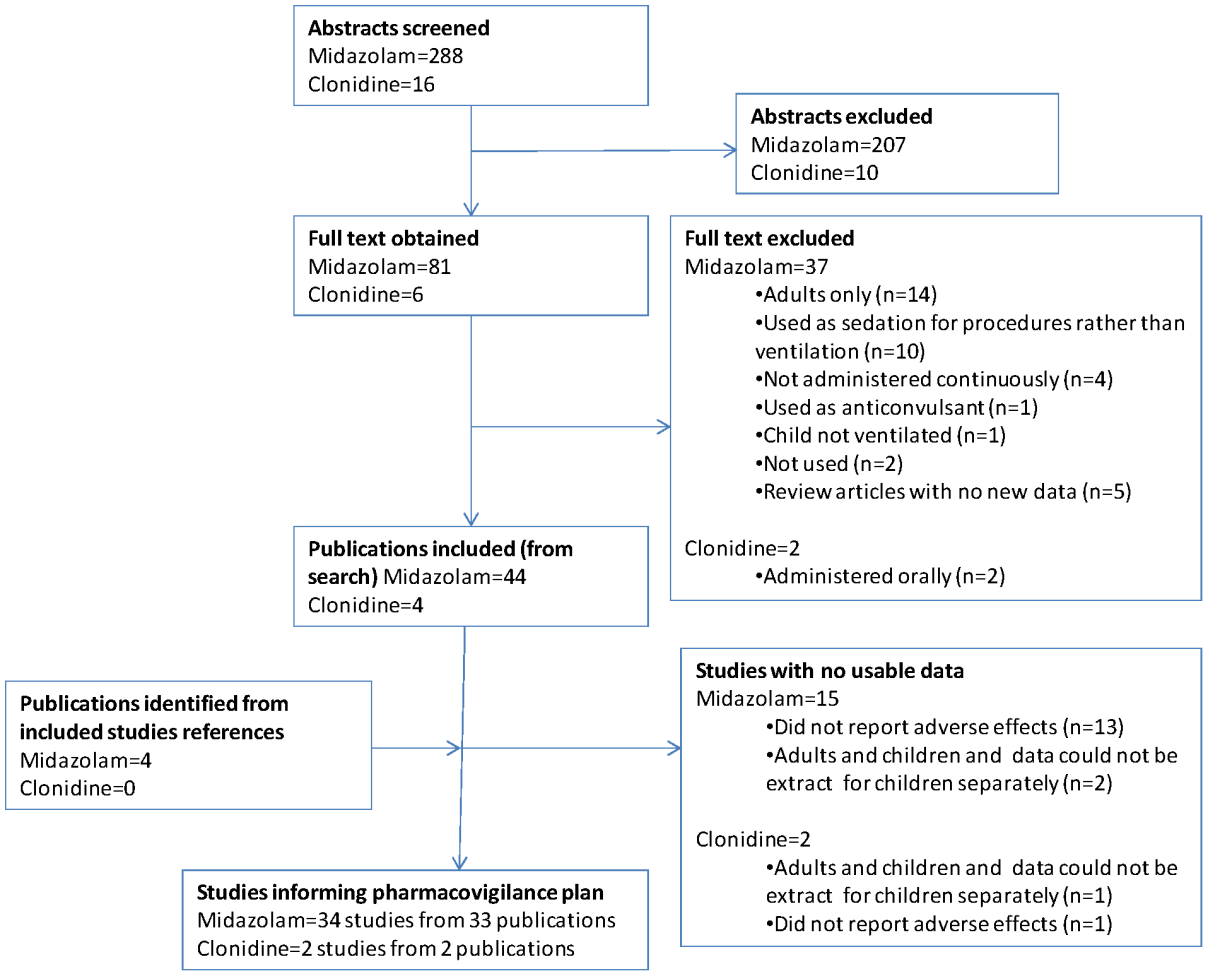
Study identification flow diagram.

The results of the search are provided in Figure 1. Table 2 gives details of excluded studies for which full texts were obtained together with descriptions of studies that did not report but may have collected relevant data.

**Table 2:** Details of excluded studies and included studies without usable data.

Category	Study	Comments
**Midazolam**		
Adults only	Aitkenhead 1989 [58]	
	Bourgoin 2003 [59]	
	Cernaianu 1996 [60]	
	Chamorro 1996 [61]	
	Costa 1994 [62]	
	Deo 1994 [63]	
	Dirksen 1987 [64]	
	Flogel 1993 [65]	
	Lee 2007 [66]	
	Lescot 2007 [67]	
	Searle 1997 [68]	
	Sinclair 1988 [69]	
	Stewart 1999 [70]	
	Tanigami 1997 [71]	
Used as sedation for a procedure rather than ventilation	Bitar 2003 [72]	This study assesses the use of sedation for plastic surgery procedures rather than as sedation on PICU
	Djurberg 2002 [73]	The study assesses anesthesia for procedures rather than sedation for mechanical ventilation.
	Hertzog 1999 [74]	This study assesses anesthesia for procedures rather than sedation for mechanical ventilation
	Hickey 1992 [75]	Midazolam not administered as a sedative in PICU but for a procedure in this study
	Koroglu 2005 [76]	Dexmedetomidine and midazolam were administered for sedation during an MRI rather than as sedation on PICU
	Laussen 1995 [77]	Although midazolam infused alongside other medications, it is for a procedure rather than a continuous infusion
	Malagon 2005 [78]	Midazolam is used as procedural anaesthesia rather than sedation
	Somri 2007 [79]	Midazolam administered for a procedure rather than as sedation and children not ventilated.
	Wilson 2003 [80]	Midazolam is used as procedural anaesthesia rather than sedation
	Yldzdas 2004 [81]	Midazolam is used as procedural anaesthesia rather than sedation
Not administered continuously	Gruber 2001 [82]	Midazolam was infused continuously during surgery but was only bolused once on PICU
	Harte 1997 [8]	Midazolam was administered by bolus rather than continuous infusion in this study
	Van Straaten 1992 [9]	Midazolam administered as a bolus rather than a continuous infusion
	Hartvig 1993 [83]	The study assessed the suitability of constant rate infusions of ketamine as a sedative agent supplemented with intermittent doses of midazolam. Not administered continuously
Used as anticonvulsant	Anand 1992 [3]	Midazolam not administered as a sedative but as a treatment for status epilepticus in this study.
Not ventilated	Prins 2005 [84]	Patient in the study were not ventilated
Not used	De Cosmo 2005 [85]	Assesses the role of propofol in Paediatric Intensive Care Unit (PICU) rather than midazolam.
	Walker 2006 [86]	Study started after midazolam ceased. This study examined the use of dexmedetomidine after the failure of the standard regimen of opioids and benzodiazepines
Review article	Aranda 2005 [15]	Review article
	Ista 2007 [16]	Review article
	Ng 2003 [10]	Review article
	Notterman 1997 [87]	Review article
	Wolf 1994 [5]	This is a commentary
No usable data	Al-Samsam 2005 [88]	This study examines the impact of environmental factors on PICU on quantity and architecture of sleep and did not assess the efficacy or safety of midazolam. 11 included patients were intubated, mechanically ventilated, and sedated with morphine and midazolam infusions.
		Likelihood of relevant safety data is low with low risk of outcome reporting bias.
	Ambrose 2000 [51]	The study assesses the adverse effects of clonidine. Ventilated children were given a background infusion of midazolam combined with a variable clonidine infusion. Reports adequacy of sedation and adverse effects on cardiovascular performance. Study is included within clonidine review but considered no usable data for midazolam.
		Likelihood of relevant safety data is low with low risk of outcome reporting bias.
	Buck 2008 [89]	This study assesses the efficacy and safety of Dexmedetomidine rather than midazolam. Dexmedetomidine was started to minimize the use of midazolam before extubation or in patients who could not tolerate midazolam.
		Likelihood of relevant safety data is low with low risk of outcome reporting bias.
	DeBerry 2005 [90]	The study is a survey of pain and sedation medications in patients on extracorporeal membrane oxygenation. The survey did not ask questions relating to efficacy or safety of the drugs used. A 6 point likert scale ranging from not effective (1) to desired effect(6) was applied. This was a general score of clinician experience across patients and did not apply to individual patients.
		Midazolam was reported to be most effective drug administered.
		Likelihood of relevant safety data is low with low risk of outcome reporting bias.
	Enomoto 2006 [91]	This was a case study involving prolonged use of dexmedetomidine on failure of midazolam with fentanyl to achieve adequate sedation and did not relate to adverse effects of midazolam. Study captured data after sedation with midazolam had ceased but midazolam reintroduced as an anti convulsant.
		Likelihood of relevant safety data is low with low risk of outcome reporting bias.
	Jin 2007 [92]	This paper assesses the use of the COMFORT score. Midazolam was administered to 5 patients and inconjuction with fentanyl to 26 patients. Data on withdrawal symptoms were collected but reported for the comparison of use of the comfort or not rather than by sedatives.
		Likelihood of relevant safety is low and risk of outcome reporting bias low as sedative types were not the focus of the paper.
	Kadilak 2004 [93]	The study assesses the safety of long-term intubation rather than the safety of drugs used for sedation by a retrospective 9-year review of children who required mechanical ventilatory support for at least 7 consecutive days. Of 98 children 76 (78%) were on midazolam infusions. Study aim did not require measurement of adverse event data.
		Likelihood of relevant safety data is low with low risk of outcome reporting bias.
	Lugo 1999 [94]	The aim of the study was to assess costs in PICU and not to assess the adverse effects of midazolam. Midazolam infusion was continued until the hourly midazolam requirement was stable for at least 24 hrs. Thereafter, patients with a nasojejunal tube who were likely to require a minimum of three additional days of continuous sedation were transitioned from intravenous midazolam to enterally administered lorazepam.
		Likelihood of relevant safety data is low with low risk of outcome reporting bias.
	Playfor 2000 [95]	This study did not assess the adverse effects of midazolam, it assessed the quality of sedation with continuous intravenous midazolam and morphine with additional oral sedation using chloral hydrate and antihistamines.
		Likelihood of relevant safety data is low with low risk of outcome reporting bias.
	Rigby-Jones 2007 [96]	This study aimed to determine the pharmacokinetics of remifentanil in children requiring ventilation after cardiac surgery. Ventilated children were sedated with a fixed rate infusion of midazolam and a remifentanil infusion.
		Likelihood of relevant safety data is low with low risk of outcome reporting bias.
	Saha 2006 [97]	The retrospective study aimed to describe which drugs were being used during transfer of critically ill children by retrieval teams.
		Likelihood of relevant safety data is low with low risk of outcome reporting bias.
	Schmidt 2006 [98]	The study prospectively matched pairs of mechanically ventilated neonates under total parenteral nutrition and midazolam sedation. The aim was to evaluate fentanyl side effects on the neonatal bladder. All 40 patients received midazolam then one group received continuous fentanyl infusions aswell with the other group serving as controls.
		Reported use of the COMFOPRT and Hartwig scores indicates measurement of efficacy. Paper reports potential gastrointestinal side effects. Likelihood of relevant data for efficacy and safety is high but risk of outcome reporting bias low as use of fentanyl on the gall bladder and gastrointestinal side effects were aim of the paper.
	Vernon 2000 [99]	This study assessed the effects of neuromuscular blockade drugs on oxygen consumption and energy expenditure. All patients were sedated using continuous infusions of midazolam and/or fentanyl.
		Likelihood of relevant safety data is low with low risk of outcome reporting bias.
Adults and children and data could not be extract for children separately	Prause 2000 [100]	Although children were included in this study, they were not analysed as a separate group therefore we are cannot determine if any adverse effects occurred in children. The authors were contacted to obtain paediatric specific data. No response received.
	Shelly 1991 [101]	Although children were included in this study, they were not analysed as a separate group therefore we are cannot determine if any adverse effects occurred in children. The authors were contacted to obtain paediatric specific data. No response received.
**Clonidine**		
Administered orally	Artman 1983 [102]	Clonidine was administered orally rather than intravenously
	Tanaka 1995 [103]	Clonidine was administered orally rather than intravenously
Adults and children and data could not be extract for children separately	Prause 2000 [100]	Although children were included in this study, they were not analysed as a separate group therefore we are cannot determine if any adverse effects occurred in children. The authors were contacted to obtain paediatric specific data. No response received.
No usable data	Jenkins 2007 [6]	The aim of this study was to investigate the current practice of sedation, analgesia, and neuromuscular blockade in critically ill children on paediatric intensive care units. The study is included in the midazolam review but excluded for clonidine. It reports 12 patients received i.v. clonidine, and it is likely that withdrawal symptoms were recorded but not reported due to the small size of the clonidine sample. The paper reports on the use of clonidine to treat withdrawal symptoms.

### MIDAZOLAM

Thirty-four studies from 33 publications were eligible for inclusion and provided usable data [6], [19], [20], [21], [22], [23], [24], [25], [26], [27], [28], [29], [30], [31], [32], [33], [34], [35], [36], [37], [38], [39], [40], [41], [42], [43], [44], [45], [46], [47], [48], [49], [50](See Figure 1).

The MHRA provided details of 10 reports of suspected ADRs.

### CLONIDINE

Two studies from two publications were eligible for inclusion and provided usable data. [51], [52]

The MHRA provided details of three reports of suspected ADRs.

### Characteristics of included studies

Summary details of eligible studies which did not report measuring adverse events are provided in Table 2.

### RCTs and CCTs

No RCTs were identified for clonidine. Seven RCTs were identified for midazolam [19], [20], [21], [22], [23], [24], [25], of which 5 were set on Neonatal Intensive Care Units [19], [20], [22], [23], [25], and two on Paediatric Intensive Care Units [21], [24]. In all but two of the studies [19], [20] patients received midazolam and concomitant opiates, either as continuous infusion or on an intermittent basis. Table S1 describes the study characteristics.

One CCT of clonidine set on a Paediatric Intensive Care Unit was identified [51] and is summarised in Table S2.

### Observational studies

Seventeen cohort studies, 13 prospective [6], [26], [27], [28], [29], [30], [33], [34], [36], [37], [39], [40], [50] and four retrospective [31], [32], [35], [38], were identified for midazolam. One study was based on a NICU [36] and the others were based on PICU. In all but three of these studies [32], [34], [37] midazolam was administered in conjunction with opiates and/or other sedative drugs. The characteristics are summarised in Table S1.

No observational studies were identified for clonidine.

### Case series and case reports

Six case series [44], [45], [47], [48], [49] and 4 single case reports [41], [42], [43], [46] were identified for midazolam. Only two of the children described in all the case reports received midazolam without concomitant opiate sedative therapy [47], [48]. See Table S3.

One case report [52] was identified for clonidine and is described in Table S2. This case was reported as clonidine was being used for a new indication.

### Information received from the MHRA

Five of the ten midazolam cases stated the duration of treatment which ranged from one day to 280 days but the information is unclear and suggests midazolam may have been administered as a bolus rather than a continuous infusion for some or all of the cases. In four of the cases, concomitant medications were administered alongside midazolam.

The following suspected adverse drug reactions were reported:

- two convulsions,
- two myoclonus,
- one tremor and dyskinesia,
- one hypoxia,
- one psychomotor, withdrawal, confusional state and hallucination,
- one withdrawal and dystonia,
- one reported asthenia, muscle disorder and myoglobinuria and
- one hepatic failure.

Three reports were received for clonidine. The duration of treatment was 1098 for the first case and one day for the following two cases. The infusion rate was unknown for the first case and 1 mcg/kg/hour and 0.7 ug/mg/hour in the other two cases. In one of the three cases, clonidine was administered with a concomitant muscle relaxant.

The following suspected adverse drug reactions were reported:

- one hypertension,
- one hypoventilation
- one cardiac arrest and bradycardia.

The value of the data provided by the MHRA is limited as we do not have any information regarding the specific age groups involved, we do not know how many, if any, children were ventilated, and we do not know what duration this data has been collected over.

### Methodological assessment of the studies

#### Risk of bias in RCTs

Table S4 provides details of the risk of bias assessment for each RCT. In summary across RCTs:

- methods of sequence generation
  - adequate [20], [21], [22]),
  - unclear [19], [20], [22], [23], [25].
- allocation concealment
  - adequate [22], [23], [24], [25]
  - Unclear (15, 16, 17).
- Masking
  - Adequate [19], [20], [22], [23], [25]
  - Unclear [21]
  - Inadequate [24] and judged to be at high risk of bias

### Quality of adverse effect monitoring

Table S4 provides details of the quality of adverse effect monitoring for each study with a summary across studies below.

### Cardiovascular

Five RCTs actively monitored cardiovascular adverse effects of midazolam [19], [20], [21], [22], [25]. Of these two described their methods for monitoring haemodynamic parameters adequately [19], [25]. One study defined haemodynamic parameters a priori in physiological terms [19] and 2 defined hypotension in terms of whether patients required inotropic or volume support [22], [25]. Three of the studies reported all cardiovascular effects numerically by treatment group [19], [21], [25]. In one study [21] all patients who received midazolam were included in the safety analysis for cardiovascular adverse effects, in one study not all patients were included [19] and in the remaining studies this information was unclear.

Four prospective cohort studies actively monitored for cardiovascular adverse effects of midazolam, and the methods are clearly described [26], [33], [40], [50]. Of these, one [26] clearly defined tachycardia a priori but the other authors did not define abnormal haemodynamic parameters a priori.

Two retrospective studies assessed cardiovascular adverse events for midazolam, but it is neither clear how rigorously these were recorded in the medical case notes, nor how thoroughly they were sought by the investigators [35], [36]. A further two cohort studies, one prospective [30] and one retrospective [37], report on cardiovascular adverse events for midazolam but it is unclear whether these were monitored by active surveillance.

The CCT [51] actively monitored cardiovascular adverse effects of clonidine and described methods for haemodynamic assessment clearly. The study reported all cardiovascular effects numerically by treatment group but excluded two patients from one of the treatment groups as there was a failure to maintain adequate sedation.

### Withdrawal

Of the 7 RCTs only 2 continued after the cessation of midazolam [23], [24] but did not actively monitor infants for signs of withdrawal. One [24] did not specify monitoring patients for signs of withdrawal, but did monitor for abnormal behaviour following cessation of the drug.

Five [6], [26], [27], [28], [34] prospective cohort studies actively monitored signs of withdrawal following cessation of midazolam. The methods used were clearly described, and three studies clearly defined the symptoms which would be considered suggestive of withdrawal [26], [28], [34].

Two other prospective cohort studies [29], [30] monitored for signs of withdrawal and three other studies [31], [37], [38] retrospectively assessed medical case notes for signs of withdrawal, but the methods were are not clearly described, and in the case of the retrospective studies it is unclear how thoroughly signs of withdrawal were documented in the notes in the first instance.

The identified CCT did not actively monitor infants for signs of withdrawal from clonidine infusion.

### Neurological

Three RCTs actively monitored patients for neurological complications while receiving midazolam [22], [24], [25]. Of these only one study [24] described the methods used adequately, and this study was also the only one to define abnormal neurological findings a priori, describe neurological adverse effects numerically by treatment group, and specifically state that all patients who received midazolam were included in the safety analysis for neurological adverse effects.

Many studies that evaluated the risks of suffering withdrawal after midazolam infusions also assessed neurological symptoms as part of this assessment. Only two [35], [38] report on children with neurological symptoms not in the context of weaning or discontinuation of midazolam. Because these were retrospective case note reviews it is unclear how well the clinical data were recorded in the first instance. One neonatal study [23] assessed neurological outcomes in preterm infants, but it would appear that this assessment was related to the clinical efficacy of sedative regimes rather than direct consequences of using midazolam infusion.

The identified CCT [51] did not actively monitor patients for neurological complications while receiving clonidine infusion.

### Prolonged sedation

One RCT [24] and two prospective cohort studies [34], [50] actively monitored children for prolonged sedation after discontinuation of midazolam infusion.

### Metabolic/endocrine

One cohort study [40] prospectively assessed children receiving midazolam for altered hypothalamic-pituitary-adrenal axis function, and the authors clearly describe the cortisol stimulation test used. One study retrospectively assessed patients for metabolic acidosis and lipaemia [32]. This was done to assess the safety of propofol rather than midazolam.

### Adverse events

#### Cardiovascular/respiratory

Two RCTs [19], [25] described some cardiovascular complications in children receiving midazolam infusion. Van Alfen van der velden [19] classed the cardiovascular complications as an ADR whereas Jacqz-Aigrain [25] did not attribute causality of the complications to the drug. One other RCT [20] also described some cardiovascular adverse effects, but classed them as not clinically significant and ‘transient’. Arya [22] and Tobias [21] did not observe adverse cardiovascular effects associated with midazolam infusion.

Van Alfen van der velden [19] described a decrease in cerebral blood flow after starting midazolam. Hypotension occurred in 7/21 preterm neonates who had received midazolam, and two of these infants required haemodynamic support. Jacqz-Aigrain [25] compared cardiovascular effects of midazolam compared with placebo in newborn infants and found that ‘heart rate and blood pressure were significantly lower in the midazolam group’, a difference which remained statistically significant until 48 hours. By day 5 the mean heart rate and blood pressure values were equal. 8 infants in the midazolam group required haemodynamic support [25]. The authors of this study did not classify these cardiovascular events as ADRs.

Three prospective cohort studies [26], [33], [36] describe some cardiovascular adverse effects of midazolam. Ista [26] states that hypotension was observed in “>13% of assessments” during weaning or after discontinuation of midazolam. Jacqz-Aigrain [36] describes hypotension in four newborn infants, in three of whom it occurred immediately after the initial bolus of midazolam, and in the fourth immediately after receiving a dose of fentanyl [36]. Shekerdemian [33] describes ‘transient’ haemodynamic changes in children receiving midazolam infusion [33]. One case report [46] describes a 40 month old child on continuous midazolam and opiate infusion who “experienced several episodes of hypotension” that required haemodynamic support.

One other case report [42] and two case series [44], [48] describe increase in heart rate and blood pressure associated with withdrawal from midazolam, rather than during the infusion. Biswas [43] reports a 6 month old child with investigations suggestive of myocardial ischaemia associated with tachycardia and hypertension from ‘Severe Benzodiazepine and Opioid Withdrawal’.

Of the seven studies that reported cardiovascular adverse effects, six of the studies appeared to classify the effects as being related to midazolam whereas one did not [25].

The CCT [51] did not observe any adverse cardiovascular effects associated with clonidine infusion and stated that “bradycardia and hypotension were not recorded in any patient”.

The case report [52] stated that “haemodynamic parameters were not adversely affected” by clonidine infusion.

### Withdrawal and behavioural

Withdrawal from midazolam was not monitored or reported in any of the 7 RCTs.

Six prospective cohort studies report possible withdrawal symptoms after continuous infusion with midazolam [6], [26], [27], [28], [34] and all were considered to be ADRs. Ista [26] reports that, in 79 patients who received midazolam infusion as sedation and were observed a total of 2188 times, over 10% of the observations suggested some symptom associated with withdrawal. Jenkins [6] reported that 29/182 infants and children receiving midazolam suffered from withdrawal symptoms as judged by the attending clinicians. The symptoms were not described. It is unclear how many of the 27 children participating in the study of weaning protocols by Ducharme [27] suffered from withdrawal, but ‘many’ of the patients had ‘behavioural distress score’ of greater than zero while weaning from midazolam. Franck [28] reports that 13/15 patients exhibited some signs of withdrawal over three or more assessment periods, and the commonest symptoms were hyperpyrexia, ‘sleeplessness’, diarrhoea, dilated pupils and tremors. The commonest symptoms were those of agitation (57/79 patients), inconsolable crying (38/79), motor disturbance (43/79), sleep disturbance (73/79), and tachypnoea (72/79). It is unclear how many patients experienced no symptoms of withdrawal at all, nor how many of the observational periods were asymptomatic of withdrawal symptoms. Sheridan [30] reports that one child suffered withdrawal symptoms after discontinuation of midazolam. These symptoms consisted of vomiting, tremulousness and sweating. Hughes [34] reports that 8/53 children in their study had ‘abnormal behaviour’ after discontinuing midazolam therapy. Three patients had visual hallucinations (one of these also had auditory hallucinations), three were ‘clearly disorientated’ and two patients did not recognise their parents, had ‘puppet-like’ movements and ‘laughed inappropriately’.

Three retrospective cohort studies report possible withdrawal symptoms. Rosen [37] reported that 1/55 patients developed visual hallucinations and tremors after discontinuing midazolam but the authors did not class this as an ADR. Bergman [38] describes 3 children who, upon discontinuation of midazolam infusion, developed neurological symptoms. The authors state that these may have been a “toxic reaction or withdrawal reaction to prolonged intravenous infusion of midazolam”. Fonsmark [31] reported that 12/38 patients receiving midazolam were retrospectively judged to be suffering from withdrawal symptoms indicating that the authors suspect the adverse effects are related to the drug.

Four case series and five case reports described symptoms that were attributed by the authors to benzodiazepine withdrawal. The symptoms reported included irritability, agitation, restlessness or inconsolability [42], [43], [44], [46], [47], [48], [49], abnormal movements (Choreoathetoid or non purposeful [42], [46], [47], seizures [49], ‘moving limbs vigorously’[44], myoclonic jerks and orofacial abnormal movements [41]), hallucinations [41], [49], grimacing [44], [49], ‘jitteriness’[44], [45], clonus [42], disorientation or abnormal communicative skills [41], [44], [46], blindness [46], abnormal behaviour [41], hyperactivity and aggression [49] vomiting [44], [48], diarrhoea [43], poor feeding [44], hypertension and tachycardia [43], [44], [48], and yawning [43].

Of the 9 observational studies that reported withdrawal symptoms, 8 of these attribute the symptoms to the drug whereas one does not [37]. In addition there were 9 case series/case reports that reported withdrawal symptoms and all attribute the symptoms to the drug. None made formal causality assessments but all the symptoms reported as withdrawal are all expected side effects of midazolam. No author used methods to distinguish whether patients were suffering from Benzodiazepine Withdrawal Syndrome, or whether their clinical features reflected withdrawal from another drug.

Neither of the studies monitored or reported withdrawal from clonidine infusion.

### Neurological

In two RCTs no neurological complications were reported [22], [24], and in one RCT [25] a child was withdrawn because of “major neurological disorders within 24 hours of inclusion, but the authors do not state that this was due to midazolam. One RCT [19] did not actively monitor participants for neurological complications, but reported that 5/11 patients treated with midazolam developed myoclonus.

Two retrospective cohort studies [35], [38] described neurological complications of midazolam infusion. Bergman [38] describes 3 children who developed abnormal movements after stopping midazolam (mentioned in section on withdrawal). One child became irritable and developed “arching of the back, stiff and abnormal movements, an inability to swallow, poor visual following, no social interaction, a stiff posture, and small amplitude choreic movements of the hands, feet and tongue”. Another child developed “choreoathetotic movements of the head, face, tongue and extremities”. The final child developed “frequent dyskinetic movements of the mouth”. The authors state that they suspected that these adverse effects “represented either a toxic reaction or a withdrawal reaction to prolonged intravenous infusion of midazolam”. Sheridan [35] reported that 2/24 children, upon extubation, developed persistent disconjugate gaze that lasted 5 days in one patient and 14 days in the other. The authors imply that they consider this to be an ADR.

Neither of the studies monitored or reported any neurological complications of clonidine infusion.

### Prolonged sedation

One RCT [24] and two prospective cohort studies [34], [50] evaluated ‘prolonged sedation’ after midazolam. Parkinson [24] did not report any prolonged sedation after discontinuing midazolam, Hughes [34] reported that 4/53 patients took between 6 hours to 1 week to become fully alert, and Lloyd Thomas [50] reports that two children suffered from ‘prolonged sedation’, lasting 3.3 hours and one lasting 20.5 hours.

### Endocrine/metabolic

One prospective cohort study (Booker 1986) assessed the effect of midazolam on the hypothalamic-pituitary-adrenal axis, by measuring response to synacthen stimulation. The authors conclude that “cortical secretion was not inhibited” by midazolam infusion [40].

One retrospective case-control study [32] compared the incidence of metabolic acidosis and lipaemia in children treated with midazolam with those treated with propofol. 17/92 patients on midazolam developed “clinically significant metabolic acidosis” and 1/92 had lipaemic serum. This primary aim of this study was to assess known side effects of propofol rather than midazolam. No other studies assessed these side effects in children treated with midazolam.

## Discussion

We have systematically reviewed the adverse effects of midazolam and clonidine in children, when used for children requiring sedation while receiving mechanical assisted ventilation on PICU. To our knowledge this is the most comprehensive systematic review to date covering a broad range of adverse effects in all paediatric age groups.

### Main findings of the review

The findings of this systematic review suggest that midazolam infusion may be associated with cardiovascular adverse effects. In neonates, midazolam has been associated with systemic and cerebral hypotension [19], . In older children an association between midazolam and haemodynamic side effects has also been suggested [46]. It would appear that discontinuation of midazolam can cause hypertension and tachycardia, which may be clinically significant [42], [43], [44], [48]. Discontinuation of midazolam has also been strongly associated with a variety of clinical features which could be suggestive of withdrawal. These symptoms can broadly be categorised as being related to irritability, neurological and behavioural abnormalities, gastro-intestinal dysfunction and autonomic dysfunction. [6], [26], [27], [28], [31], [34], [37], [38], [42], [43], [44], [45], [46], [47], [48], [49]. It would also appear, unsurprisingly, that these problems become more likely in children receiving higher doses of midazolam over longer periods [6], [31]. It is difficult to accurately estimate the frequency with which withdrawal symptoms occur, but in the prospective studies that we identified the reported incidence appears to range from 15% [6], [34]to 85%[28].

Midazolam infusion is also associated with neurological side effects [35], [38] and prolonged sedation [34], [40].

There are a limited number of studies published regarding the use of clonidine in all paediatric age groups. The studies we have reviewed would suggest that clonidine infusion does not have adverse cardiovascular effects [51], [52].

### Robustness of this review

The scope of our review was focussed enough to enable us to concentrate on one specific indication of midazolam and clonidine, but broad enough for us to feel that we have included as much information about the side effects of these drugs when given in this situation as possible. Our review was conducted according to a predefined protocol, which was designed according to the guidelines suggested by the Cochrane Adverse Effects Methods Group [53]. In doing so we feel we have not only systematically identified all the relevant literature, but also rigorously appraised it.

Midazolam and clonidine are not only used for sedation on PICU. Clonidine is also administered orally for preoperative sedation in the paediatric population. Midazolam can also be administered as an intravenous bolus, as a subcutaneous infusion, intranasally or via the buccal route. The sedative properties of midazolam can be utilised during operative procedures, or for simple procedures on general paediatric wards, while its anticonvulsant properties render it a useful therapy for status epilepticus. These alternative indications and preparations of midazolam and clonidine may be associated with adverse effects specific to these settings and uses and warrant a separate systematic review of safety. It was felt that the safety of continuous infusion of midazolam and clonidine as sedation on PICU is a hugely important and distinct clinical question, which we feel justifies our exclusion of studies relating to other uses of these drugs.

### Quality of the evidence

The studies we identified varied significantly in quality, and the data regarding adverse effects that we have identified must be interpreted in the context of a variety of limiting factors.

Much of the data in our review has come from observational studies and case reports. This is especially evident with regard to the paediatric (rather than neonatal) age group. For example, the only information relating to cardiac side effects during midazolam in children is from a case report [46]. With regard to the evaluation of withdrawal symptoms associated with midazolam, the data are all derived from observational studies in older children. One of the RCTs [24] we included was not adequately masked, and as the outcomes measured in this trial were subjective, this may have biased the results.

None of the studies included in this review used a causality tool to assess the likelihood that the adverse events identified were ADRs. However, all the adverse effects that we identified are already listed in the respective Summary of Product Characteristics and can therefore be considered “expected”.

Consensus guidelines regarding the conduct of clinical trials of sedation for neonates have been produced, but there is currently no consensus for the methodology, definitions, or outcomes that should be used in infants and older children on PICU [54], [55]. The studies we have identified vary in terms of the rigour with which adverse effects are sought, and the analysis and reporting of the results. The tools used to evaluate the presence of withdrawal, and the definitions of what constitutes benzodiazepine withdrawal itself, also vary between studies. Furthermore, the tools which are currently available have not been sufficiently validated, and this may explain the non-uniformity with which they are used in clinical research [56]. The clinical features of Benzodiazepine withdrawal are similar to those from opiate withdrawal, stress, delirium and inadequate pain management, and this already makes assessment of these symptoms within the context of the studies we have identified very difficult [5], [26]. Until a measure of withdrawal that has been properly designed and rigorously evaluated for measures of validity, reliability, responsiveness and ease of use in clinical and research situations the identification and quantification of benzodiazepine withdrawal in children will be compromised.

### Implications for pharmacovigilance assessment in the SLEEPS trial

The decision about whether to collect all adverse event data or restrict collection should be determined by a risk assessment for the trial and consider how well established the risk/benefit profile of the medicines under study are, licensing status of the drugs and current level of clinical use. [57] The risk assessment for the SLEEPS trial was also informed by this systematic review which did not identify any additional safety concerns to those specified within the existing SmPCs therefore a decision was made to restrict data collection to adverse reactions. This decision was also influenced by the administrative burden at sites if research nurses were requested to collect all adverse events occurring in critically ill children, and that the volume of events unrelated to treatment would reduce the focus of pharmacovigilance monitoring. However given the low levels of data relating to clonidine we aimed to collect all adverse reactions rather than restrict to serious adverse reactions. In addition given the relatively sparse data on withdrawal symptoms this was added as a secondary outcome of the trial. This was considered particularly important as younger children may require substantially higher doses (mg/kg) than older children and adolescents and the risk of dependence is known to increase with dose and duration of sedation. Specifying withdrawal as an outcome meant that symptoms would be actively assessed in all children. The pharmacovigilance plan was specified within the trial protocol and reviewed by the MHRA during the CTA assessment.

In conclusion, our review has highlighted significant and potentially serious side effects associated when midazolam is administered according to usual PICU practice. Because children on PICU are already critically ill, side effects due to midazolam can easily be over-looked due to other pressing issues with their care or can be attributed to other causes of cardiovascular or neurological disturbance associated with their disease or treatment. This may explain the relatively low number of reports held by the MHRA. It is therefore crucial that clinicians are aware of the potential iatrogenic risks of midazolam and consider this drug as a potential cause of adverse events and actively report suspected reactions to the MHRA. Conversely the evidence regarding the safety of clonidine infusions as sedation for ventilated children on PICU is sparse. Critically ill children need adequate sedation to protect these vulnerable children from the known adverse effects of withholding sedation during their PICU admission. Identifying the specific features of drug side effects, finding an optimum drug therapy at the appropriate dose and achieving the right balance of sedation is a major challenge. Finding this balance requires high quality clinical research to improve what is currently a poor evidence-base regarding safety and optimal delivery.

## Supporting Information

Table S1 (PDF)

Table S2 (PDF)

Table S3 (PDF)

Table S4 (PDF)
