# Supplementary material for: The Role of Systematic Reviews in Pharmacovigilance Planning and Clinical Trials Authorisation Application: Example from the SLEEPS Trial
Source: PLoS One. 2013 Mar 15;8(3):e51787. doi: 10.1371/journal.pone.0051787 (PMC3598865; doi:10.1371/journal.pone.0051787)
Supplement: Table S1 — (PDF) [file pone.0051787.s001.pdf]

Table S1 Characteristics of Randomised Controlled Trials, Controlled Clinical Trials and observational studies.

Study	Design	Population	Follow up/ duration of treatment	Dosing regime
				**Loading dose (LD) and Continuous infusion rate (CIR)**
Arya 2001[22]	RCT<br>Midazolam (n=17) vs placebo (n=16)	Preterm neonates (mean gestation age: 31.5 weeks<br>All infants were also receiving opiate infusion	48 hours of follow-up<br>Duration of ventilation median=53 hrs Range=(7 to 216)	LD= 200 mcg/kg<br>CIR=60 mcg/kg/hr
Anand 1999[23]	RCT<br>Midazolam (n=22) vs morphine (n=24) vs placebo (n=21)	Preterm neonates (24-32 week gestational age; < 72 hours postnatal age<br>Patients received opiates if required for analgesia/sedation	Study drug infusions were continued for as long as necessary or a max of 14 days<br>Ventilator support mean 14.2 hrs SD=11.1<br>Patients monitored until 12 hours after discontinuation of drug	LD=200 mcg/kg<br>CIR=20, 40 or60 mcg/kg/hr depending on gestation age
Jacqz-Aigrain 1994[25]	RCT<br>Midazolam vs placebo	24 Preterm Neonates (mean gestational age: 32.1; < 48 hours postnatal age)<br>Patients were allowed to receive bolus doses of opiates if required, but not continuous infusion	Patients monitored for 5 days of treatment but not to or following extubation<br>Mean duration of treatment mean=78.7 hrs SD=30.9 hrs across midazolam and placebo groups	LD=Not administered<br>CIR=30 or 60 mcg/kg/hr depending on gestational age
Parkinson 1997[24]	RCT<br>Midazolam vs Chloral hydrate and promethazine	22 (1 day to 17 years)<br>Both groups also received morphine infusion	Patients monitored after treatment cessation until patient breathing spontaneously<br>Durations of treatment and lengths of follow up not described	LD= Not administered<br>CIR=50 mcg/kg/hr starting rate. 300mcg/kg/hr maximum rate
Tobias 2004[21]	RCT<br>Midazolam (n=10) vs	Infants (2 to 70 months)<br>Patients received opiates if required for	Patients followed for 48 hours of treatment of which only 24 hours was midazolam.	LD=Not administered<br>CIR=100 mcg/kg/hr

	Dexmedetomidine 0.25 mcg/kg/hr (n=10) vs Dexmedetomidine 0.5mcg/kg/hr.	analgesia/sedation	After 24hrs on midazolam or dexmedetomidine if mechanical ventilation was still necessary, the patient was switched to the alternative and the study continued<br>Midazolam infusion duration mean=22hrs SD=8hrs	
Treluyer 2005[20]	RCT<br>All infants (n=23) were treated with midazolam and different doses compared	Preterm neonates<br>(>33 weeks gestation age; <28 weeks post-natal age)<br>Patients did not receive opiates	Infusion continued for up to 48 hours<br>Median observation time period 12h (1-60)	LD=75 - 200 mcg/kg<br>CIR= Each patient randomised to 37.5, 50, 62.5, 75, 87.5 or 100 mcg/kg/hr
Van Alfen van der Velden 2006[19]	RCT<br>Midazolam (n=11) vs morphine (n=10)<br>Patients treated with midazolam did not receive opiates	Preterm neonates<br>(26-33 week gestational age)	Outcomes measured up to 120 mins after start of sedation only.	LD= 200 mcg/kg<br>CIR= 200 mcg/kg/hr
Bergman 1991[38]	Retrospective Cohort (n=45)	mean=3.94 years range=(0.03 years to 19.2 years)<br>Patients also received opiates	Duration of treatment: Mean infusion time 128 hours (range 12-917)<br>Length of follow up not described.	Maximal dose received: 230mcg/kg/hr (70-940)<br>Mean Total dose received: 29.8mg/kg (1.4-286)
Booker 1986[40]	Prospective cohort (n=50)	(6 months to 9 years)<br>All children were also administered morphine	Duration of treatment: Mean=46.7hr Range=(13-197hrs) SD=54<br>Length of follow up post extubation was not	Mean dose: 228mcg/kg/hr<br>Maximal dose received: 564mcg/kg/hr

			described	
Ducharme 2005[27]	Prospective cohort<br>(n=27)	(> 1 month to 19years)<br>Opioids were also administered to some patients	Duration of treatment: Range: <1 day, >21 days<br>Length of follow up post weaning not provided	Not provided
Fonsmark 1999[31]	Retrospective cohort (n=38)	(6 months to 14 years)	Duration of treatment:<br>Withdrawal group: 4 days (range 1-13)<br>Non-withdrawal group: 3 days (range 1-11)<br>Length of followup post treatment not described	*Withdrawal group:* total dose 30.9 mg/kg (0.01-99.2)<br>*Non-withdrawal group*: total dose 4.6 mg/kg (0.3-33)<br>All children received 'analgesics, mainly morphine'
Franck 2004[28]	Prospective cohort (n=13)	(6 weeks to 28 months)<br>All children also received opiates	Duration of treatment prior to tapering median=9 days range=4-18<br>Duration of followup 2 days after treatment discontinuation	Cumulative dose median 26.4 mg/kg (range 0-154).
Hartwig 1991[39]	Prospective cohort<br>(n=24)	(26 days to 5 years)<br>All patients were also administered fentanyl	Duration of treatment: mean =11.6 days range= (38hr-40 days)<br>Length of follow up post infusion was not described.	Doses ranged from 50-400mcg/kg/hr
Hughes 1994[34]	Prospective cohort<br>(n=53)	(6 days – 11 years)<br>Patients did not receive concomitant opiates	Duration of treatment: range 22 – 429 hrs<br>Duration of follow up: max of 1 week post	Maximal dose received (range) 25-720mcg/kg/hr

			patient being fully conscious in presence of withdrawal symptoms	
Ista 2008[26]	Prospective cohort (n=79)	Median age 3.4 months; range 0 days to 15.5 years<br>73 patients also received opiate infusion	Duration of treatment: Median 10 days (range 3-108)<br>Withdrawal symptoms observed median 6 days (1 to 67)	Median continuous dose 176 mcg/kg/hr (range 25-397); Median maximal dose 300 mcg/kg/hr (range 25-700)
Jacqz-Aigrain 1992[36]	Prospective cohort (n=15)	neonates<br>(29-41 weeks gestational age)<br>7 of the patients also received opiates	Duration of treatment: mean= 60 hrs SD=23.3<br>Samples taken up to 24hr post infusion	CI= 60mcg/kg/hr
Jenkins 2007[6]	Prospective cohort (360 children on PICU of which 268 were mechanically ventilated. 186/268 received midazolam)	(Median age 1.4 years, IQR0.33-7)<br><br>Other sedative/analgesic agents were also used.	Duration of treatment: not provided<br><br>Followup until PICU discharge median 3 days IQR 1-6 days	*Withdrawal group:* Median dose<br>2mg/kg/day (IQR 1.1-3.3)<br>*Non-withdrawal group:* median dose 1 mg/kg/day (IQR 0.5-1.9)
Lloyd-Thomas 1986[50]	Prospective cohort (n=10)	(6 months to 8.75 years)<br>All children were also administered morphine	Duration of treatment: Mean=23.4 SEM= 0.69<br><br>Length of follow up post extubation was not described	Mean dose: 186mcg/kg/hr<br>Maximal dose received: 300mcg/kg/hr
Pepperman 1997[32]	Retrospective Cohort (propofol v non-propofol)	(9 days to 16 years)	Duration of treatment: Range 13 minutes to 11 days	Mean dose: 400mcg/kg/hr (range 10-4900)

	(n=61)	"Most patients" received supplementary opiates"	Length of followup not described	
Rosen 1991[37]	Retrospective Cohort (n=55)	(0-19 years)<br><br>Supplementary opiates were prescribed as required	Duration of treatment: Median: 74hrs Range=(4-1272)hrs<br>Length of follow up post infusion was not described	Mean dose: 55.2mcg/kg/hr (-/+32.4)<br>Maximal dose received: 240mcg/kg/hr
Shekerdemian 1997[33]	Prospective Cohort (n=10)	(Median age 3.3 years<br><br>Range: 0.4–13.6)<br>All patients were also sedated with opiate infusion.	Duration of treatment: not provided<br><br>Length of follow up not described	Range: 100-200mcg/kg/h
Sheridan 1994 [35]	Retrospective Cohort (n=24)	(7 months to 17 years)<br>All patients were also treated with opiates	Duration of treatment: Mean= 16.5 days range=(4-56)<br><br>Length of followup post extubation not described	Mean maximal dose received: 110mcg/kg/hr range (40-350)
Sheridan 2001[30]	Prospective cohort (n=28)	(6 months to 16 years)<br>All patients also received opiates	Duration of treatment: Median: 15 days range 8-112<br><br>Range: (1.1 – 48.9)<br>Length of follow up post extubation not described.	Median dose: 160mcg/kg/hr<br>Dose range: 30mcg/kg/hr – 290mcg/kg/hr
Sheridan 2003[29]	Prospective cohort	(1.2 -13 years)<br>Opioids were also administered	Average duration: 12.7+/-10.9 days<br>Range: 2 – 33 days	Average dose: 0.15+/-0.16mg/kg/hr

	(n=11)		Length of follow up post extubation not described	Dose range: 0.06-0.65 mg/kg/hr
For characteristics of case reports and case series of midazolam see table 5				
