# Supplementary material for: The Role of Systematic Reviews in Pharmacovigilance Planning and Clinical Trials Authorisation Application: Example from the SLEEPS Trial
Source: PLoS One. 2013 Mar 15;8(3):e51787. doi: 10.1371/journal.pone.0051787 (PMC3598865; doi:10.1371/journal.pone.0051787)
Supplement: Table S2 — (PDF) [file pone.0051787.s002.pdf]

Table S2 Characteristics, quality and safety of clonidine studies.

Non randomised controlled trials				
**Study**	**Overview**	**Safety**	**Efficacy**	**Quality**
Ambrose 2000[51]	**Population:**<br><br>30 ventilated children aged 10 years and under.<br><br>**Interventions:** Group 1: 10 unparalysed ventilated patients were administered a clonidine infusion of 0.2 -1.0 mcg/kg/hr.<br><br>Group 2: a further 10 unparalysed ventilated patients were administered a clonidine infusion of 0.2-2mcg/kg/hr.<br><br>Group 3: 10 postoperative cardiac surgical patients were administered a fixed infusion rate of 1.0mcg/kg/hr.<br><br>All patients were administered a background dose of midazolam	**Cardiovascular/respiratory:** There were no "adverse effects on cardiovascular performance" in the two groups of unparalysed ventilated patients (group 1 and 2). The postoperative cardiac surgical patients (Group 3) had no significant change over 6 hours in heart rate, blood pressure or derived cardiac index.<br><br>"Bradycardia and hypotension were not recorded in any patient"<br><br>Withdrawal: not assessed<br><br>Neurological: not assessed	Group 1: Two patients failed to maintain adequate sedation at the maximum infusion of clonidine 1 mcg/kg/hr, but the other 8 patients were adequately sedated for the entire study period.<br><br>Group 2: Adequate sedation was recorded in 602 of the 672 hourly assessments (89.5%) and there were no failures.<br><br>Group 3: Not described	**Ascertainment of AE data** Cardiovascular: actively sought. Methods for haemodynamic assessment clearly described.<br><br>Withdrawal: Not assessed<br><br>Neurological: not assessed<br><br>**Reporting of AE data:** Data presented numerically by intervention group. 2 patients were withdrawn from group 1 as there was a failure to maintain adequate sedation. Data up until withdrawal were included in the safety analysis for these patients.
**Case reports/case series**				

Study	Case series/case report	Summary	Adverse effects
Lyons<br><br>1996[52]	Case report	11 year old boy who suffered second and third degree burns to 78% of his body was intubated and ventilated using synchronised intermittent mandatory ventilation. Clonidine was introduced intravenously for pain relief after a rise in morphine requirements at a rate of 7.5 to10mcg every 4 hours	When the rate of clonidine infusion was increased from 7.5 to10mcg every 4 hours haemodynamic parameters were not adversely affected.
