# Supplementary material for: The Role of Systematic Reviews in Pharmacovigilance Planning and Clinical Trials Authorisation Application: Example from the SLEEPS Trial
Source: PLoS One. 2013 Mar 15;8(3):e51787. doi: 10.1371/journal.pone.0051787 (PMC3598865; doi:10.1371/journal.pone.0051787)
Supplement: Table S3 — (PDF) [file pone.0051787.s003.pdf]

Table S3 Characteristics and safety of midazolam case reports and case series.

Study	Summary	Adverse effects
Bergman 1991[38]	**Case 1**: A 5-month-old girl admitted with rapid respiratory rate. Chest x-ray showed hyperaeration and a right middle lobe infiltrate. Previous medical history unremarkable. Sedated from day 2 to day 7 with iv midazolam and fentanyl. Dose not provided.<br><br>**Case 2**: 15 month old girl with Down syndrome and asthma, with a surgically repaired complete atrial-ventricular septal defect with a cardiac arrest at 6 months of age during surgical repair. Admitted with wheezing and respiratory distress but without cyanosis. Sedated for 10 days with iv midazolam and fentanyl. Dose not provided.<br><br>**Case 3**: 3 month-old girl admitted for a repair of a left anomalous coronary artery. Previous medical history included moderate mitral regurgitation and congestive heart failure, and a respiratory syncytial virus upper respiratory tract infection 1 week before admission. Sedated for 5 days following surgery with iv midazolam and fentanyl. Dose not provided.	**Case 1:** after discontinuation the child remained poorly interactive with the environment and exhibited irritability, with a high-pitched cry, arching of the back, stiff and abnormal movements, and an inability to swallow. Neurologic examination 10 days after admission revealed poor visual following and no social interaction. 5 weeks after admission neurological exam was normal.<br>**Case 2:** child did not look at objects or interact socially with the environment and was in constant abnormal motion. 6 weeks after admission she had returned to normal but was not crawling or using a pincer grasp as prior to hospitalisation. This was resolved 5 months after admission.<br>**Case 3:** she did not display her previous developmental abilities. She followed movement only briefly, did not smile, coo or grasp, and was having unusual movements of her tongue and extremities. She lay in a stiff posture with the left arm and left leg flexed, the right arm and leg extended, and both hands fisted. 2 weeks after admission the infant was normal.
Biswas 2005 [43]	A 6-month old female born at 25 weeks gestation with severe opioid and benzodiazepine dependence resulting from multiple operative procedures and chronic ventilatory support received continuous intravenous infusion of fentanyl and midazolam.<br><br>Midazolam was infused at a rate of 0.2mg/kg/hr	**Cardiovascular:** After 5 days of midazolam infusion tachycardia (190-200bpm, baseline 130bpm) and hypertension (120-130 systolic/70-80 diastolic). Her peripheral perfusion remained normal except for central capillary refill time of 3 seconds. 12 lead ECG showed significant ST depression in the anterior leads. Cardiac enzymes were also raised.

		**Withdrawal/neurological:**<br>At the same time as the cardiovascular problems arose she acutely developed agitation, loose stools, and yawning.
Carnevale 1997 [44]	The authors describe 5 children who received midazolam. All of whom also received infusions of opiates<br>**Case 1**: 11month old girl who had undergone a surgical repair of tetraology of Fallot.<br><br>**Case 2**: 7 month old boy who sustained a skull fracture and a cerebral infusion.<br><br>**Case 3**: A 16-month old girl who had second- and third-degree burns on 30% of her body surface.<br><br>**Case 4**: A 2 month old boy with bronchiolitis.<br><br>**Case 5**: A 5 month old girl with bronchiolotits.<br><br>Midazolam was infused at a rate of 240mcg/kg/hr to 1.44mg/kg/hr. I.44mg/kg/hr was administered inadvertently to one and apart from this maximum infusion was 300mcg/kg/hr.	**Cardiovascular:** An elevated heart rate was documented in the 7 month old boy within 13 hours of the first decrease in the midazolam infusion.<br><br>**Withdrawal/neurological:**<br><br>**Case 1:** Inconsolable crying was noted within 24hours following the decrease of fentanyl and the discontinuance of midazolam.<br><br>**Case 2:** Inconsolable crying and vomiting (along with severe coughing and an elevated heart rate were documented within 13 hours of the first decrease in the midazolam infusion. Poor feeding appeared and the severity of inconsolable crying increased, after discontinuance of both fentanyl and midazolam. Jitteriness was noted 12 hours after the cessation of both drugs.<br><br>**Case 3**: Inconsolable crying and grimacing were reported following a decrease of midazolam. 12 hours following the discontinuance of midazolam, inconsolable crying and irratibility /agititation/fussiness were reported (with significant severity) for 6 days. Grimacing was also reported for 1 day.

		**Case 4**: Previous agitation persisted and increased gradually through the 24 hours following the weaning and extubation. He also exhibited crying, moving all his limbs vigorously, not sleeping at all overnight, and poor feeding.<br><br>**Case 5**: At the first discontinuance of midazolam, the child was noted to have gagged and vomited. At the second cessation of midazolam, jitteriness, gagging and vomiting, followed by poor feeding, were noted less than 24 hours following cessation of midazolam, along with an increased severity of agitation.
Cho 2007 [42]	6 year old boy with severe burns requiring artificial ventilation for 29 days.<br><br>Sedated with both midazolam and fentanyl. Midazolam dose was initially 0.1 mg/kg/hr, and by day 22 the rate had increased to 0.8 mg/kg/hr (maximum dose required)	**Cardiovascular/respiratory**:<br><br>On initiation of drug: None mentioned in report<br><br>On cessation of drug:HR 144 and BP 160/101 within 48 hours of weaning midazolam and fentanyl.<br><br>Respiratory: None mentioned in report<br><br>**Withdrawal and neurological:** Within 48 hours of weaning sedatives developed abstinence syndrome. This presented as clonus, agitation and choreoathetoid movement disorder.
Ducharme 1995 [46]	40 month old child requiring mechanical ventilation was sedated with midazolam and fentanyl	**Cardiovascular:** The child "experienced several episodes of hypotension that required dopamine and dobutamine infusions to maintain perfusion".

	Midazolam dose: 30 mcg/kg/hour, increased due to tolerance to 2 mcg/kg/min. Duration of midazolam sedation: 7 days	**Withdrawal/neurological:** The child's recovery from sedation and analgesia was initially described as 'uneventful'. He complained to his parents of temporary blindness on the day midazolam was stopped. He became agitated on day 8 and required sedation with chloral hydrate. On day 8 he was unresponsive and unable to recognise his parents. For the next 2 days he was unresponsive, had 'non purposeful movements' and was globally aphasic. He also had thrombocytosis which 'coincided with his worse cognitive and motor status'. This lasted for a week, and after one month rehabilitation and treatment with midazolam and fentanyl his motor function improved but his speech remained limited.
Epstein 2007 [41]	Previously healthy 4 year old boy ventilated for 4 days after developing epiglottitis.<br><br>Sedated with midazolam and fentanyl. Midazolam dose was 0.5 mg/kg/hr	**Cardiovascular/respiratory**:<br>None mentioned in report<br><br>**Withdrawal and neurological:** within hours of extubation the child appeared alert but did not respond to verbal commands. He exhibited intermittent tongue protrusion, inappropriate laughter and lipsmacking behaviour. There were occasional truncal myoclonic jerks. He also had visual hallucinations and other orofacial dyskinesias. This syndrome resolved over 4 days
Sury 1989 [49]	3 children (aged 4 years, 11 years and 12 years) sedated with midazolam and opiate infusions whilst receiving mechanical ventilation. Midazolam doses: 120 to 240 mcg/kg/hr.<br>4 year old: mean dose 220 mck.kg/hr, Maximal dose 370 mcg/kg/hr, 7	**Cardiovascular:** None reported

	days duration of sedation<br><br>11 year old: mean dose 170 mcg/kg/hr, maximal dose 375 mcg/kg/hr, 14 days duration of sedation<br><br>12 year old: mean dose 560 mcg/kg/hr, maximal dose 1 mg/kg/hr, 17 days duration of therapy	**Withdrawal/neurological/ behavioural:**<br><br>4 year old patient: 24 hours after stopping midazolam became hyperactive and aggressive, and uncommunicative. He was also disorientated and suffered from hallucinations. He required 7 days of diazepam to provide relief from these symptoms<br><br>11 year old girl developed visual hallucinations and suffered two episodes of generalised convulsions within 24 hours of discontinuing midazolam.<br><br>12 year old girl: became agitated, uncommunicative and abusive 24 hours after stopping midazolam. She also developed facial grimacing. She required IV diazepam to treat these symptoms
Tobias 1994 [47]	The authors describe 2 children requiring ventilation who received midazolam infusions as sedative therapy. One was a 17 month old child with viral pneumonia, and the other a 15 month old with subglottic stenosis.<br><br>Midazolam dose:<br>15 month old- 0.1 mg/kg/hr to 0.4 mg/kg/hr;<br><br>17 month old- 0.3 mg/kg/hr	**Cardiovascular:** none mentioned in report<br><br>**Withdrawal/neurological:** The 15 month old is described to have become irritable and inconsolable after withdrawal of midazolam and other sedative agents. He was also noted to have choreoathetoid movements of his upper limbs. These neurological problems responded to treatment with Lorazepam<br><br>The 17 month old child was diagnosed with withdrawal from barbiturates. The authors do not describe withdrawal from midazolam in this patient.

Van Engelen 1993 [48]	The authors describe 2 children who had been artificially ventilated and sedated with midazolam. The first child was 15 months old (Midazolam dose: 214 mcg/kg/hr, duration of initial sedation 29 days) and the second was 14 days old (Midazolam dose:285 mcg/kg/hr, duration of initial sedation 12 days)	**Withdrawal/cardiovascular:** The 15 month old child had received midazolam as a continuous infusion for 12 days, but not opiates. Within 'half a day' of stopping the drug, he became restless, tachycardic and hyperpyrexic. He also vomited repeatedly. These symptoms resolved within 30 minutes of restarting the midazolam. Over the next 11 days the midazolam was stopped twice, and on both occasions the same clinical features occurred.<br><br>The 14 month old baby had received midazolam for 29 days while ventilated. He also received nicomorphine. Within 12 hours of discontinuing the drug he became restless, developed a distended stomach secondary to aerophagia and began vomiting. The symptoms resolved within 12 hours of restarting midazolam. 5 days later his midazolam was stopped again, and he was extubated. 12 hours later, however, he became restless, developed tachycardia and started vomiting, which resulted in aspiration pneumonia. He was reintubated, and midazolam restarted. Within 12 hours the symptoms disappeared.
Yaster 1996 [45]	A four month old infant required Extra Corporeal Membrane Oxygenation, then mechanical ventilation, after cardiac surgery. He was sedated with continuous infusion of fentanyl and midazolam. Upon cessation of these drugs he developed jitteriness which was treated with Lorazepam.	**Cardiovascular:** Not discussed<br>**Withdrawal:** the child developed 'jitteriness' during weaning of midazolam
