# Supplementary material for: The Role of Systematic Reviews in Pharmacovigilance Planning and Clinical Trials Authorisation Application: Example from the SLEEPS Trial
Source: PLoS One. 2013 Mar 15;8(3):e51787. doi: 10.1371/journal.pone.0051787 (PMC3598865; doi:10.1371/journal.pone.0051787)
Supplement: Table S4 — (PDF) [file pone.0051787.s004.pdf]

Table S4 Quality and safety of Midazolam RCTs and observational studies.

Randomised controlled trials			
**Study**	**Safety**	**Efficacy**	**Quality**
Anand 1999 [23]	**Cardiovascular/respiratory**: None mentioned in report<br><br>**Withdrawal:** Two neonates from the morphine group developed mild opioid withdrawal". There is no report of infants in the midazolam group suffering from withdrawal.	Sedation scores were not significantly altered from baseline in any groups. In the midazolam group, the COMFORT score was 15.9 (SD3.8) before drug and 14.9 (SD 4.6) during drug administration. In the morphine sulphate group the COMFORT score was 17.3 (SD 4.6) before drug and 14.7 (SD 3.2) during drug administration. In the dextrose group, the COMFORT score was 15.6 (SD 3.2) before drug and 17.5 (4.2) during drug administration.<br><br>Overall clinical outcomes: Poor neurological outcomes in 24% of placebo group, 32% in midazolam group and 4% in morphine group.	**Assessment of bias:**<br><br>Sequence generation: Low risk<br>Allocation concealment: Low risk<br>Blinding: Low risk<br><br>**Ascertainment of AE data:**<br><br>Cardiovascular: No discussion in study methods<br>Withdrawal: No mention in methods, although this is mentioned in the discussion: "all neonates were assessed with the Finnegan Neonatal Abstinence Scale at 12 and 24 hours (and then daily) after discontinuation of treatment with the study drug.<br><br>**Reporting of AE data:** It is unclear whether all infants were included in the safety analysis
Arya 2001[22]	**Cardiovascular/respiratory:**	At 18 hours 13/14 children in the	**Assessment of bias:**

	Midazolam and placebo groups were 'comparable for hemodynamic variables' over the study period'. Heart rate, blood pressure and perfusion status were not different between the two groups. No infants developed hypotension after receiving midazolam.<br><br>Measures of oxygenation, ventilator parameters and blood gases remained similar between the two groups<br><br>**Withdrawal:** Not assessed<br><br>**Neurological:** No patients in the midazolam group were specifically reported to have developed 'epileptiform movements'.<br><br>However 2 patients in the placebo group developed this problem 24 hours after enrolment into the trial.	midazolam group were adequately sedated compared with 8/14 in the placebo group. At 24 hours 14/14 children in the midazolam group were adequately sedated compared with 9/13 in the placebo group.	Sequence generation: Low risk<br>Allocation concealment: Low risk<br>Blinding: Low risk<br>**Ascertainment of AE data:**<br>Cardiovascular: actively sought. Methods of measuring haemodynamic parameters not described. Definition for hypotension/bradycardia not given in physiological terms. However the authors do say that they monitored "haemodynamic instability (hypotension, tachycardia, oliguria) which would require volume expansion and/or vasoactive drugs"<br><br>Withdrawal: Not assessed<br><br>Neurological: The presence of epileptiform movements was actively monitored. Method of monitoring not described.<br><br>**Reporting of AE data:**

			Cardiovascular: Heart rate is reported numerically for the value taken immediately post-bolus, but descriptively for the remainder of the readings. Blood pressure itself is not reported at all, but the authors state that “after bolus of Midazolam or placebo none developed hypotension” and “the groups were comparable for their perfusion status and urine output” for the 48 hours after starting the infusion. It is unclear whether all babies were included in the safety analysis.<br><br>Neurological: The authors state that 2 babies in the placebo group developed epileptiform movements, but do not specifically mention whether any babies in the Midazolam group developed these problems or not.
Jacqz-Alrgain 1994 [25]	**Cardiovascular:** Heart rate and blood pressure were significantly lower in the Midazolam group	“Continuous infusion of midazolam at doses adapted to gestational age induces effective	**Assessment of bias:**<br>Sequence generation: Unclear

	than placebo group. These were significantly different after 24 and 48 hours ($p<0.01$ and $p<0.05$ respectively). Although mean heart rate and systolic blood pressure remained lower until day 5, the differences were not statistically different between day 2 and day 5. At day 5 the mean values were equal.<br><br>Haemodynamic instability requiring inotropic support and/or volume expanders occurred in 8 babies from the midazolam group, and 6 from the placebo group<br><br>There were no differences between the groups in terms of oxygenation, ventilator support, chronic lung disease, necrotising enterocolitis or death.<br><br>**Withdrawal:** Not assessed<br><br>**Neurological:** One baby in the midazolam group was withdrawn because of 'major neurological disorders within 24 hours of inclusion'	sedation in newborn babies". In the midazolam group adequate sedation was present in 75 – 100% of babies during treatment whereas in the placebo group adequate sedation was present in 26 – 45% of babies during treatment.	Allocation concealment: Low risk<br><br>Blinding: low risk (Medical staff and trial personnel blinded)<br><br>**Ascertainment of AE data:**<br>Cardiovascular: Actively sought. Methods of haemodynamic assessment described. Hypotension not defined in physiological terms, but the authors express the result as the number of patients with haemodynamic instability (hypotension, tachycardia, oliguria) requiring plasma volume expanders and/or vasoactive drugs.<br><br>Withdrawal: Not assessed<br><br>Neurological: actively sought. Method of exactly who monitored the patients and how often is not described.<br><br>**Reporting of AE data:**<br>Cardiovascular: Haemodynamic variables and presence of haemodynamic instability are

			presented numerically for all groups. Unclear whether all babies included in safety analysis<br><br>Neurological: data on epileptiform movements not reported
Parkinson 1997[24]	**Cardiovascular/respiratory**:<br><br>None mentioned in report<br><br>**Withdrawal:** Not assessed<br>**Neurological/behavioural:**<br>No children exhibited abnormal behaviour after midazolam administration.<br><br>**Prolonged sedation:** No patient suffered from 'prolonged sedation' after midazolam	Midazolam appeared to be less effective than chloral hydrate/ promethazine at sedating children requiring mechanical ventilation. In the chloral hydrate/promethazine group 61% of sedation assessments were classified as satisfactory whereas in the midazolam group 48% of sedation assessments were classified as satisfactory.	**Assessment of bias:**<br><br>Sequence generation: Low risk<br>Allocation concealment: Low risk<br>Blinding: Unclear. Method of blinding not described. It is possible that outcomes could be affected by this<br>**Ascertainment of AE data:**<br><br>Cardiovascular: not assessed<br>Withdrawal: not assessed<br>Neurological/Behavioural:

			Actively sought. The methods used to assess abnormalities in behaviour are not described in this paper, but a reference for the methods used is given (Hughes 1994). Methods are clearly described here. The definition of 'abnormal behaviour' is not given in this paper, but is discussed in the other reference. The definition of 'prolonged sedation' is not given a priori.<br>**Reporting of AE data:**<br>The data relating to abnormal behaviour and prolonged sedation are described numerically for the two groups. All children who were randomised were included in the safety analysis.
Tobias 2004 [21]	**Cardiovascular/respiratory:** There were no	36 morphine boluses were	**Assessment of bias:**

	differences between the three treatment groups with regard to blood pressure. There were no adverse haemodynamic events (bradycardia or hypotension) in the midazolam group. Heart rate was significantly higher in the midazolam group (mean HR 142 bpm) as compared with Dexmedetomidine groups (mean HR 122 and 112)<br><br>**Withdrawal:** Not assessed	administered as rescue medication to the midazolam group, compared to 29 and 20 boluses administered to the 0.25 mcg/kg/hr Dexmedetomidine and 0.5 mcg/kg/hr Dexmedetomidine groups respectively. Total supplemental morphine required in midazolam group was 0.74 mg/kg/24 hours compared to 0.55 mcg/kg/24h and 0.28 mck/kg/24h in 0.25 mcg/kg/hr Dexmedetomidine and 0.5 mcg/kg/hr Dexmedetomidine groups respectively	Sequence generation: unclear<br>Allocation concealment: unclear<br>Blinding: unclear – it is unclear whether medical caregivers or trial personnel were blinded to intervention groups<br><br>**Ascertainment of AE data:**<br><br>Cardiovascular: actively sought. Method of assessment of haemodynamic parameters not described.<br><br>Bradycardia/hypotension not defined in the methods<br><br>Withdrawal: not assessed<br><br>**Reporting of AE data:**<br><br>Cardiovascular: data for BP/HR are presented
Treluyer 2005 [20]	The authors state that “no serious adverse event was reported during the study”<br><br>**Cardiovascular/respiratory:** Within one hour of	Estimated probability of baby receiving adequate sedation was 76.9% for the group receiving 200 micrograms/kg loading dose	**Assessment of bias:**<br><br>Sequence generation: Unclear<br>Allocation concealment: unclear

	starting midazolam, there was a decrease in median blood pressure values of 2% (systolic BP), 7% (diastolic BP) and 6% (Mean Arterial BP). A decrease of >30% was noted in systolic BP in no patients, in diastolic BP in 2 patients, and Mean ABP in 1 patient. All these changes were described as 'very transient', and no patient required haemodynamic support. Relative reduction in heart rate was 4%. 2 patients developed pneumothorax.<br><br>**Withdrawal:** Not assessed		Blinding: Low risk (nurses and doctors blind to the dose of midazolam)<br><br>**Ascertainment of AE data:**<br><br>Cardiovascular: actively sought. Method of assessment of haemodynamic parameters not described. Bradycardia/ hypotension not defined in methods. Also 'transient' not defined a priori in methods<br><br>Withdrawal: Not assessed<br>**Reporting of AE data:**<br><br>Cardiovascular: Data presented numerically. Unclear whether all 23 infants were included in the safety analysis. HR and BP reported at one hour, but subsequent measurements at 4,12,18,24 and 48 hours not reported
Van Alfen-van der	**Cardiovascular/respiratory:** After initiating	Not assessed	**Assessment of bias:**

Velden 2006 [19]	midazolam infusion there was a decrease in the mean cerebral blood volume and cerebral flow velocity. There was a decrease in mean peripheral oxygen saturation and MABP. In 7 infants hypotension was observed - occurring within 15 minutes. One of these required inotropic support and one required plasma expanders.<br><br>Decreases in arterial and transcutaneous oxygenation and cerebral blood oxygenation index were observed in 5 patients. These changes occurred within 5 minutes of starting midazolam. Two patients required increase in Fi02 and 1 required increase in PIP. These changes occurred in 6 patients treated with morphine. There was no significant change in blood gas values within 2 hours of administering midazolam.<br><br>**Withdrawal:** Not assessed		Sequence generation: unclear<br>Allocation concealment: unclear<br>Blinding: Low risk<br><br>**Ascertainment of AE data:**<br><br>Cardiovascular: actively sought.<br><br>Methods for haemodynamic assessment clearly described. Definition for ‘hypotension’ given. Other measurements defined.<br>Withdrawal: Not assessed<br><br>Neurological: Not described in study methods. Myoclonus not described or defined in results<br><br>**Reporting of AE data:**<br><br>Cardiovascular: Data presented numerically by intervention group. 2 patients in each group

	**Neurological:** 5/11 patients treated with midazolam suffered from myoclonus. One of these patients was also hypocalcaemic		excluded from analysis of cerebral blood flow data because of technical problems. All patients included in the other analyses.

**Observational Studies**			
**Study**	**Safety**	**Efficacy**	**Quality**
Bergman 1991 [38]	**Cardio vascular:** None described<br><br>**Withdrawal/neurological:**<br><br>5 children had possible symptoms of decreased responsiveness, tongue thrusting, staring and shaking. These were "in the days after midazolam was stopped". 40 children had no symptoms. 3 had definite symptoms. In one child this presented as poor interaction with the environment, irritability, a	Not assessed.	**Ascertainment of AE data:**<br>Cardiovascular: not assessed<br><br>Withdrawal/neurological: Retrospective analysis. It is unclear whether these were monitored at the time of recording the medical notes.

	high-pitched cry, arching of the back, stiff and abnormal movements, an inability to swallow, poor visual following, no social interaction, a stiff posture and small-amplitude choreic movements of the hands, feet and tongue. In the second child, this presented as not looking at objects, no social interaction with the environment and was in constant abnormal motion. When awake the child had constant choreoathetotic movements of the head, face, tongue and extremities. The third child did not display her previous developmental abilities, followed movement inconsistently and briefly, did not smile, coo or grasp, had frequent dyskinetic movements of the mouth, including pursing and chewing movements and rapid, repetitive tongue thrusting and had stiff posture.		Reporting: Reported numerically and descriptively.
Booker 1986 [40]	**Cardiovascular/respiratory:**<br><br>The authors state that “at no time was any change in cardiovascular variables, or the need for cardiovascular support, attributed to the infusion of midazolam”.<br><br>**Withdrawal:** Not assessed<br>**Endocrine:** The authors state that “cortisol secretion was not inhibited by this sedative regime”.<br><br>**Local:** One patient displayed an area of redness around the infusion site but no other local complications were observed.	The authors state that “clinically adequate sedation was obtained in 47 patients (94%)”.	**Ascertainment of AE data:**<br>Cardiovascular: Actively sought. Methods of monitoring haemodynamic parameters described. Hypotension not defined.<br>Withdrawal: Not assessed<br><br>Endocrine: Method of assessment described.<br>**Reporting of AE data:**<br><br>Cardiovascular: Data not presented numerically, but is presented descriptively

			Endocrine: Mean cortisol levels presented before and after synacthen stimulation
Ducharme 2005 [27]	**Cardiovascular:** none mentioned in the report<br>**Withdrawal:** The rates of withdrawal are unclear but it would appear that several patients had a behavioural distress score of more than zero whilst weaning.	Not assessed.	**Ascertainment of AE data:**<br><br>**Cardiovascular:** Not assessed<br><br>**Withdrawal/neurological:** Monitored prospectively. Methods of monitoring and recording described but the score used is unvalidated. "Behavioural distress" is not defined so significance of the score is not clear.<br>**Reporting:** All the patients are described and their maximum behavioural distress score is described numerically.
Fonsmark 1999 [31]	**Cardiovascular:** None mentioned in study report<br>**Withdrawal:** 12/38 patients who received midazolam were judged to be suffering from withdrawal	Not assessed	**Ascertainment of AE Data:**<br><br>Cardiovascular: Not assessed

			Withdrawal: Actively sought in notes. However it is not clear whether the symptoms of withdrawal were actively monitored at the time of discontinuation of the drug. Symptoms of withdrawal that were sought in the notes are defined.<br><br>**Reporting of AE data:**<br><br>Withdrawal: data presented numerically (ie number of patients suffering from withdrawal)
Franck 2004 [28]	15 patients underwent 693 assessments of withdrawal (2 patients did not receive midazolam).<br><br>Cardiovascular: none mentioned in the report	Not assessed	**Ascertainment of AE data:**<br><br>Cardiovascular: not assessed<br><br>Withdrawal: monitored

	**Withdrawal:** Thirteen children exhibited signs of withdrawal on at least 3 assessments. The commonest symptoms, which occurred in "over one third of assessments when patients were experiencing withdrawal" were temperature>37.2 ^{o}C, 'sleeplessness', diarrhoea, dilated pupils and tremors		prospectively. Methods clearly described. A scoring system was used that was designed for this study, and 'preliminary' validation had been performed previously. Symptoms of withdrawal clearly defined.<br><br>Reporting of AE data: All children who had received midazolam were included in the analysis. The two children who did not receive midazolam are not analysed separately from the 13 patients who received midazolam and opiates.
Hartwig 1990 [39]	The authors mention no adverse effects of midazolam that were reported during the infusion. The authors claim that no patients suffered respiratory complications after extubation and discontinuation of midazolam.	Not assessed	**Ascertainment of AE data:**<br><br>Cardiovascular: not described<br><br>Withdrawal: Not described<br><br>**Reporting of AE data:**<br><br>Data not presented numerically, but is presented descriptively

Hughes 1994 [34]	**Cardiovascular:** None mentioned in the study report<br><br>**Withdrawal:** Nine patients had abnormal behaviour after stopping midazolam. 3 of these children had visual hallucinations, and one of these also had auditory hallucinations. 3 were 'clearly disorientated' and 2 patients did not recognize their parents, had puppet-like movements and laughed inappropriately. The duration of these symptoms lasted from 3 hours to 1 week.<br>One child had a 'paradoxical reaction' to midazolam, and became agitated within 12 hours of starting the drug.<br><br>**Prolonged sedation:** 4/53 patients took 6 hours to 1 week to become fully alert.	Not assessed	**Ascertainment of AE data:**<br><br>Cardiovascular: Not assessed<br><br>Withdrawal/abnormal behaviour: Actively sought. Methods of monitoring clearly described. Definitions of withdrawal and abnormal behaviour clearly stated.<br><br>Prolonged sedation: Not defined a priori. Methods used to assess prolonged sedation are described.<br><br>**Reporting of AE data:**<br><br>Presented numerically and descriptively.
Ista 2008 [26]	**Cardiovascular/respiratory**:<br>Hypertension was observed in ">13% of assessments" during weaning or after discontinuation of midazolam. No other cardiovascular symptoms were mentioned in the study report whilst midazolam was being received.	Not assessed	**Ascertainment of AE data:**<br><br>Cardiovascular: Actively sought as part of the withdrawal checklist. The highest values for heart rate,

	**Withdrawal:** Symptoms of withdrawal were observed in ">10% of assessments", and are presented here as "Symptom (number of patients experiencing symptom)<br><br>*Central Nervous System irritability:* Anxiety (41), agitation (57), Increased muscle tension (38), Slight muscle jerks (30), Uncoordinated movements (43), Tremors in response to stimuli (11), spontaneous tremors (9), Inconsolable crying (38), high pitched crying (18), grimacing (36), sleep reduction to <1 hour (54), sleep reduction to 1-3 hours (73), Seizures (4), Pupil dilatation (14), Hallucinations (8)<br><br>*Gastrointestinal:* Diarrhoea (45), vomiting (21), Increased gastric residuals after feeding (32), poor feeding (9)		respiratory rate and arterial blood pressure were automatically generated by the patient data management system for the previous 4 hours. Tachycardia clearly defined. Hypertension only monitored in those with arterial lines.<br>Withdrawal: Actively sought using a checklist composed by the authors for this study. The checklist was "approved by ten experienced pediatric

	Autonomic dysfunction: tachycardia (53), tachypnoea (72), hypertension (42), fever (39), sweating (32), sneezing (11), yawning (23), mottling (19)		intensivists". There is no other discussion of the validation of this questionnaire. The symptoms associated with withdrawal are listed. Some (eg sleep reduction) are defined, but many (eg poor feeding, tachycardia are not). The 79 participants were observed for signs of withdrawal 2188 times (Median 14 assessments/child, range 2-198) over a median of 6 (range 1-67) days . 42% of these observations were made within 24 hours of discontinuing midazolam.<br><br>**Reporting of AE data:**<br>Withdrawal: All symptoms are reported numerically. 2 patients who would otherwise have been eligible for the review were excluded because they had 'severely disturbed behaviour pattern'.
Jacqz-Aigrain	**Cardiovascular:**	Not assessed	**Ascertainment of AE data:**

1992 [36]	Hypotension was observed in 4 children, ranging from 30 to 37 weeks gestation. In 3 of these the BP fell immediately after the initial bolus of midazolam. In the fourth patient the hypotension occurred while he was receiving an infusion, and happened immediately after a dose of fentanyl was given.<br><br>**Withdrawal:** Not assessed		Cardiovascular: Unclear whether actively or passively sought. Unclear what methods used to monitor cardiovascular AE. Hypotension not defined<br><br>Withdrawal: Not assessed<br><br>**Reporting of AE data:**<br><br>Cardiovascular: Presented numerically.
Jenkins 2007 [6]	**Cardiovascular/respiratory**:<br><br>None mentioned in report<br><br>**Withdrawal:** 34/267 intubated patients were reported to show phenomena that could be associated with withdrawal. 29/34 (85%) of these patients had received midazolam.	Not assessed	**Ascertainment of AE data:**<br><br>Cardiovascular: not collected<br><br>Withdrawal: Unsure whether data actively or passively sought. Data regarding withdrawal was prospectively collected. Investigators at participating centres did not have fixed clinical definition of what constitutes withdrawal – ie decision based on clinical judgement.<br><br>**Reporting of AE data:**<br>Withdrawal: Presented

			numerically
Lloyd Thomas 1986 [50]	In 8 children there were no adverse effects of midazolam infusion reported.<br><br>**Cardiovascular/respiratory:** The cardiovascular variables remained stable, and when patients were on CPAP, the ventilator parameters remained normal.<br><br>**Prolonged sedation:** Two children had high plasma concentrations of midazolam. One had prolonged sedation lasting 20.5 hours. The second child also had prolonged sedation, lasting 200 minutes<br><br>**Withdrawal:** Not assessed	The authors report that “satisfactory sedation was achieved in all patients”	**Ascertainment of AE data:**<br><br>Cardiovascular**:** Actively sought. Methods of monitoring haemodynamic parameters described. Hypotension not defined.<br><br>Withdrawal: Not assessed.<br><br>Prolonged sedation: Not defined<br><br>**Reporting of AE data:**<br><br>Data not presented numerically, but is presented descriptively
Pepperman 1997 [32]	**Cardiovascular/respiratory:** none mentioned in study report<br><br>**Withdrawal**: Not assessed<br><br>**Metabolic/biochemical:**<br><br>Metabolic acidosis: 17/92 (18%) patients sedated with Midazolam developed ‘clinically significant metabolic acidosis’. This is compared to 17/106 (16%) patients receiving propofol who developed the same complication<br><br>Lipaemia: One patient treated with Midazolam had lipaemic serum	Not assessed	**Ascertainment of AE data:**<br><br>Cardiovascular: not assessed<br><br>Withdrawal: not assessed<br><br>Metabolic: Metabolic acidosis Retrospectively sought in medical notes (metabolic acidosis routinely sought). ‘Metabolic acidosis’ defined

			Lipaemia; Retrospectively sought in medical notes (unclear how measured). 'Lipaemia' not defined.<br><br>**Reporting of AE:** presented numerically
Rosen 1991 [37]	**Cardiovascular/respiratory:** Blood pressure and heart rate remained within 10% of baseline values. In patients requiring inotropic support, no patients required an increase in these drugs during the midazolam infusion. No adverse respiratory effects were observed in the cohort of patients. Three patients underwent 'metabolic studies' – there was a mean 28% reduction in oxygen consumption, a 5% decrease in CO_2 production, and a 5% rise in the respiratory quotient after starting midazolam.<br><br>**Withdrawal/neurological:** One patient had hallucinations and tremors that occurred 48 hours after abrupt discontinuation of midazolam. No seizures were observed during administration of the midazolam.	Midazolam infusions were effective in sedating all the children in the study.	**Ascertainment of AE data:**<br><br>Cardiovascular: Actively sought retrospectively in medical notes. Methods of this are not described however. Hypotension/tachycardia not defined. 'Metabolic studies': Unclear whether actively or passively sought in notes. Only a proportion of patients underwent these investigations<br><br>Withdrawal/neurological: Unclear whether actively or passively sought in notes.<br><br>**Reporting of AE data:**

			Cardiovascular: Data not reported numerically
Shekerdemian 1997 [33]	**Cardiovascular:**<br><br>Cardiac output-there was a transient fall in cardiac output: after the initial bolus of midazolam the mean Cardiac Index fell from 5.1(0.5)l/min to 3.7(0.4) l/min, and after one hour were 4.6(0.4) l/min<br><br>Oxygen consumption: fell by 16.5%(2.9)% after bolus of midazolam and then rose in all but 3 patients by one hour.<br><br>Mean heart rate: no significant change after 15 minutes, but slight rise at 1 hour.<br><br>No change in right atrial pressure or left atrial pressure or systemic vascular resistance: No change. Pulmonary resistance: slight rise within first 15 minutes in 4 patients with indwelling left atrial catheters – did not reach level of statistical significance.<br><br>**Withdrawal:** Not assessed	Not assessed	**Ascertainment of AE data:**<br>Cardiovascular: Actively sought, and methods clearly described. Adverse haemodynamic effects not defined a priori.<br><br>Withdrawal: Not assessed<br><br>**Reporting of AE data:**<br><br>Cardiovascular: results reported numerically. All AE outcomes reported.
Sheridan 1994 [35]	**Cardiovascular/respiratory:**<br><br>The authors state that "No hypotension or problems weaning from mechanical ventilation were seen secondary to the use of Midazolam infusion".<br><br>**Withdrawal:** Not assessed	Not assessed	**Ascertainment of AE data:**<br><br>Cardiovascular: Retrospectively analysed from medical notes. Method of data extraction not described. 'Hypotension' not

	**Neurological:** 2 children had persistent disconjugate gaze and diminished responsiveness after extubation. This resolved spontaneously after five days in one patient, and after 14 days in the other. Both children made a complete recovery. CT scan of the head was normal.		defined.<br><br>Withdrawal: Not assessed<br><br>Neurological: Retrospective analysis from medical notes. Unclear whether actively sought in notes.<br><br>**Reporting of AE data:**<br><br>Cardiovascular: reported that patients had 'no hypotension'.<br><br>Withdrawal: Presented numerically and descriptively
Sheridan 2001 [30]	The authors state that 'all children survived to discharge and there was no perceived morbidity related to the high doses of background medication used during their acute illness'<br><br>**Cardiovascular/respiratory:**<br><br>Not specifically discussed in study report.<br><br>**Withdrawal:** One child suffered withdrawal symptoms after discontinuation of morphine and midazolam. These symptoms consisted of vomiting, tremulousness and sweating. The authors also report that 'all children were discharged without opiate or benzodiazepine medications'.	Not assessed	**Ascertainment of AE data:**<br><br>Cardiovascular: It is unclear whether haemodynamic adverse effects were actively sought, or identified by routine clinical monitoring on PICU<br><br>Withdrawal: It is unclear how the authors monitored for the presence of withdrawal symptoms. Withdrawal syndrome not defined a priori

			**Reporting of AE data:**<br>Data regarding withdrawal presented numerically.
Sheridan 2003[29]	**Cardiovascular/respiratory:**<br>Not specifically discussed in study report.<br>**Withdrawal:**<br>Authors state that "there were no withdrawal symptoms noted".	Not assessed	**Ascertainment of AE data:**<br>Cardiovascular**:** Not assessed<br>Withdrawal**:** It is unclear how the authors monitored for the presence of withdrawal symptoms. Withdrawal syndrome not defined a priori<br>**Reporting of AE data:**<br>None reported
